# Prolyl isomerase Pin1: a promoter of cancer and a target for therapy

**DOI:** 10.1038/s41419-018-0844-y

**Published:** 2018-08-29

**Authors:** Yang Chen, Ya-ran Wu, Hong-ying Yang, Xin-zhe Li, Meng-meng Jie, Chang-jiang Hu, Yu-yun Wu, Shi-ming Yang, Ying-bin Yang

**Affiliations:** 1 School of Life Science, Southwest University. No.1, Tiansheng Road, Beibei District, 400715 Chongqing, China; 2 Department of Gastroenterology, Xinqiao Hospital, Third Military Medical University. No.183, Xinqiao Main Street, Shapingba District, 400037 Chongqing, China

## Abstract

Pin1 is the only known peptidyl-prolyl *cis*–*trans* isomerase (PPIase) that specifically recognizes and isomerizes the phosphorylated Serine/Threonine-Proline (pSer/Thr-Pro) motif. The Pin1-mediated structural transformation posttranslationally regulates the biofunctions of multiple proteins. Pin1 is involved in many cellular processes, the aberrance of which lead to both degenerative and neoplastic diseases. Pin1 is highly expressed in the majority of cancers and its deficiency significantly suppresses cancer progression. According to the ground-breaking summaries by Hanahan D and Weinberg RA, the hallmarks of cancer comprise ten biological capabilities. Multiple researches illuminated that Pin1 contributes to these aberrant behaviors of cancer via promoting various cancer-driving pathways. This review summarized the detailed mechanisms of Pin1 in different cancer capabilities and certain Pin1-targeted small-molecule compounds that exhibit anticancer activities, expecting to facilitate anticancer therapies by targeting Pin1.

## Facts

- Pin1 is the only known peptidyl-prolyl *cis*–*trans* isomerase (PPIase) that regulates the conformational transformation of phosphorylated Serine/Threonine-Proline (pSer/Thr-Pro) motif.
- Pin1 is highly expressed in the majority of cancers and negatively related to the clinical prognosis.
- Pin1 facilitates multiple cancer-driving pathways.
- Pin1 is a potential target for cancer therapy.

## Open Questions

- What are the mechanisms for the high expression of Pin1 in cancer?
- How does Pin1 upregulate the oncogenes and inhibit the cancer suppressors?
- What are the molecular mechanisms of Pin1 that lead to cancer immune escape?
- How does Pin1 facilitate the tumor-promoting inflammation?

## Introduction

**Fig. 1:**
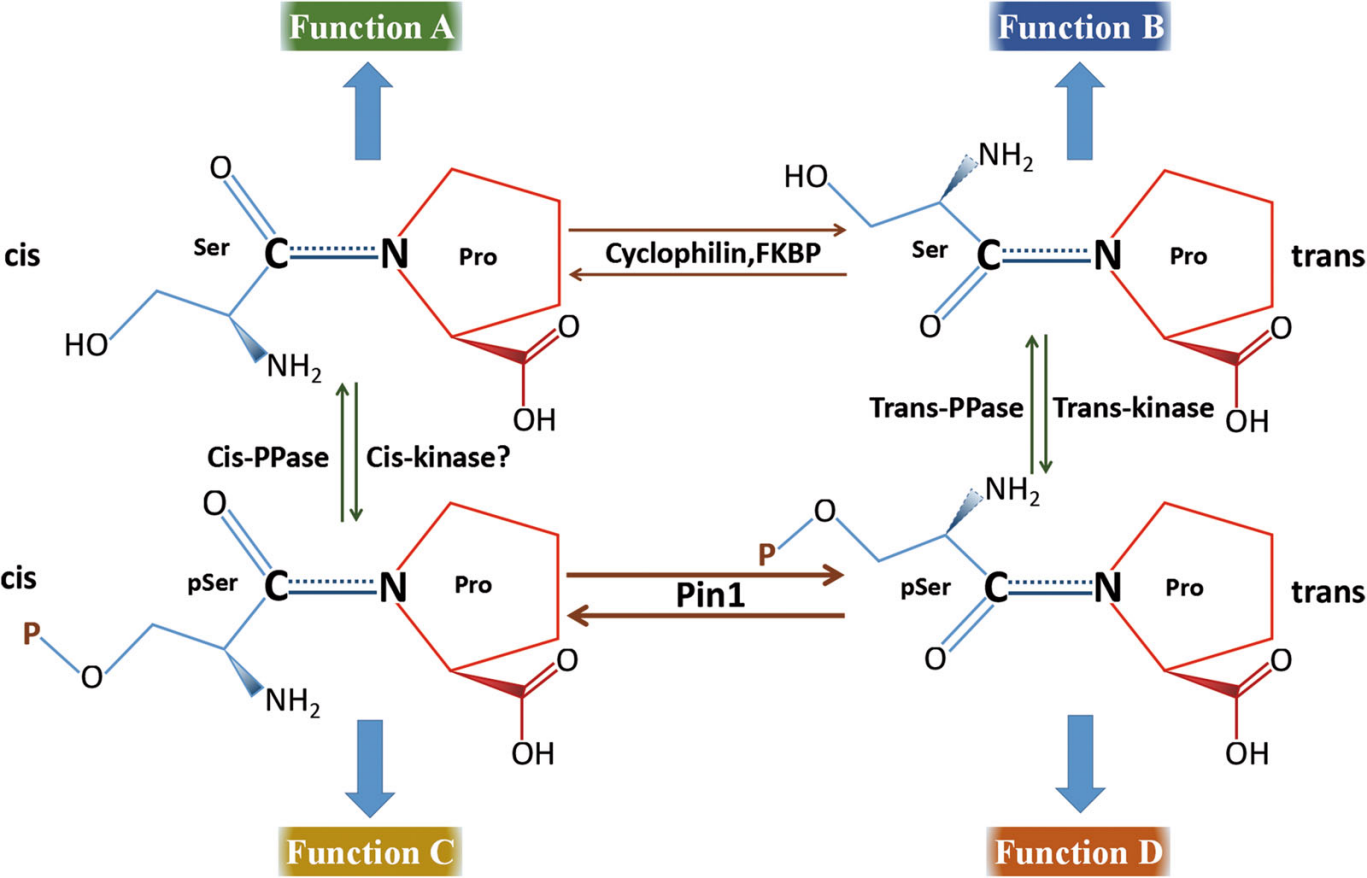
PPIases mediate structural transformation of target substrates. Peptidyl-prolyl uniquely exhibits a *cis* or *trans* conformation. Different proline-directed kinases and phosphatases specifically recognize *cis* or *trans* Ser/Thr-Pro motif to modify the function of target proteins that highlight the PPIase-mediated structural transformation. The PPIases, including cyclophilins, FKBPs, and parvulins. Cyclophilins and FKBPs mediate the turnover of unphosphorylated substrates and Pin1 mediates the isomerization of phosphorylated substrates

Proline (Pro)-directed Serine/Threonine (Ser/Thr) phosphorylation is a common modification of numerous signaling pathways. Many Pro-directed kinases, including mitogen-activated protein kinases and cyclin-dependent kinases (CDKs), are involved in this process^1–3^. Owing to the unique side-chain groups of proline, peptidyl-prolyl adopts an alterable *cis* or *trans* conformation^4^. The peptidyl-prolyl *cis*–*trans* isomerases (PPIases) accelerate the structural transformation of peptidyl-prolyl to regulate the folding, subcellular location, stability, activation, and interaction of multiple proteins^5–7^. The PPIase superfamily includes cyclophilins, FK506-binding proteins (FKBPs), and parvulins^8^ (Fig. 1). Cyclophilins and FKBPs can be inhibited by the immunosuppressants cyclosporin A (CyA) and FK506/rapamycin, respectively^8^. Pin1 belongs to parvulins and can be inhibited by juglone^9^. It is the only known PPIase that mediates the isomerization of phosphorylated Ser/Thr-Pro (pSer/Thr-Pro) motif^10^. Pin1 is comprised of an N-terminal WW domain and a C-terminal PPIase domain, which are connected by a flexible linker^11,12^.

**Fig. 2:**
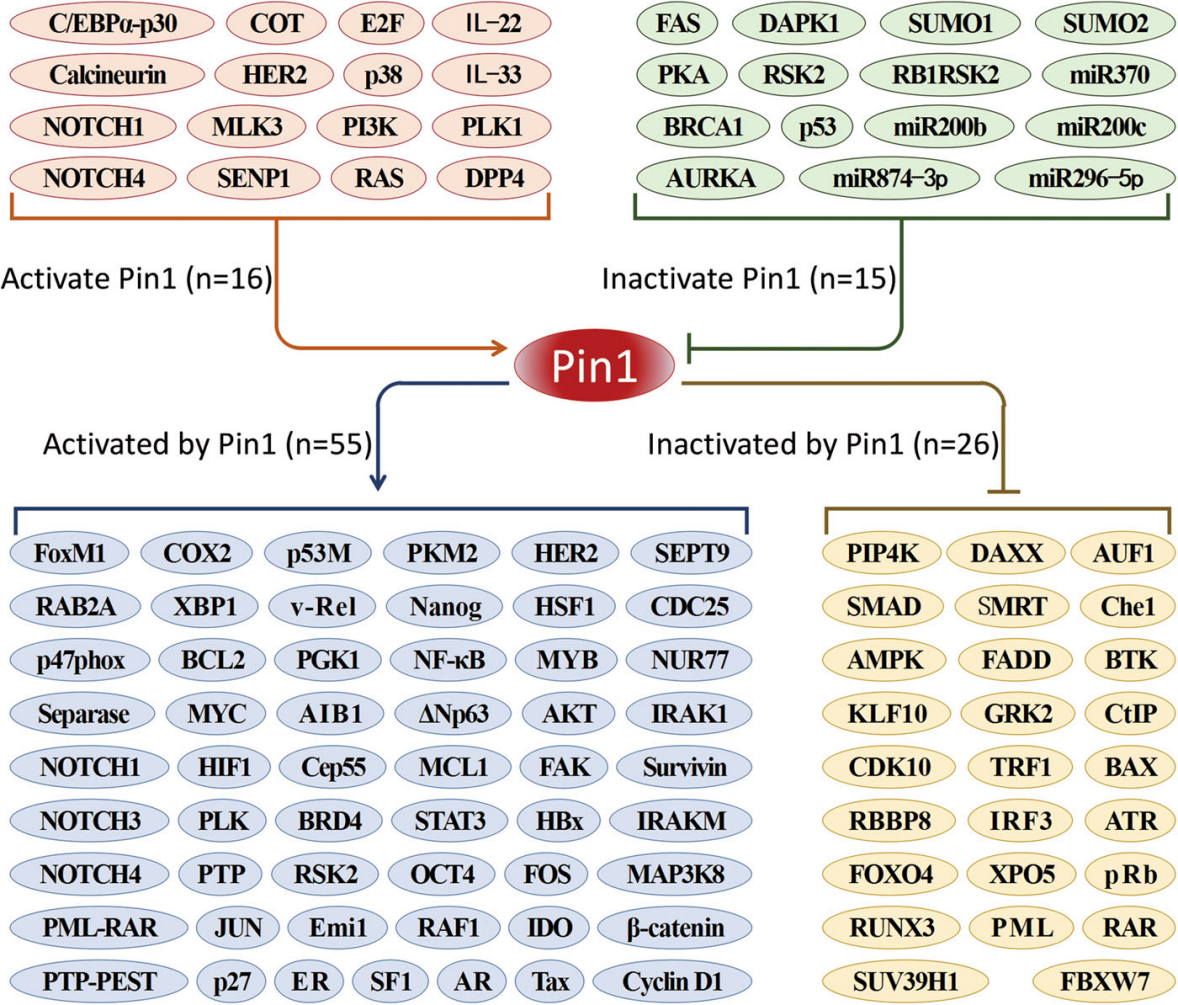
The regulators and targets of Pin1. Pin1 is generally activated by oncogenes and inactivated by cancer suppressors. Meanwhile, Pin1 upregulates >50 oncogenes or proliferation-promoting factors and downregulates >20 tumor suppressors or proliferation-suppressing factors

The expression of Pin1 is immediately regulated by transcription factors E2F^13^ and NOTCH1^14^. Beside, the CCAAT/enhancer binding protein-α (C/EBPα)-p30 increases Pin1 expression by recruiting E2F to the promoter of Pin1^15^. After that, the mRNA level of Pin1 is reduced by some microRNAs (miRNAs), including the recently discovered miR-370^16^ and miR874-3p^17^. Moreover, the posttranslational modifications of Pin1, including phosphorylation^18,19^, sumoylation^20^, ubiquitination^21^, and oxidation^22^, regulate the stability, substrate-binding ability, PPIase activity, and subcellular localization of Pin1. These processes are always aberrant in cancer that contribute to the high expression and/or overactivation of Pin1 (Fig. 2).

Pin1 is involved in multiple cellular processes, including division^23^, differentiation^24^, senescence^25^, and apoptosis^26^. Pin1 is always deficient in degenerative disorders, including Parkinson’s disease (PD)^27^, Alzheimer’s disease (AD)^28^, and Huntington’s disease (HD)^29^. In contrast, it is highly expressed in most cancers, especially in cancer stem cells (CSCs), and negatively related to the clinical prognosis^30–32^. The depletion of Pin1 significantly inhibits tumorigenesis in the mice models that are derived by mutation of p53^33^, activation of HER2/RAS^34^ or constitutive expression of c-Myc^35^. Additionally, many Pin1-targeted inhibitors, including all *trans* retinoic acid (ATRA)^36^, juglone^37^, and KPT-6566^38^, have showed cancer suppression ability in multiple researches (Table 1).

**Table 1:** Pin1 inhibitors

PIN1 inhibitor	Chemical structure	Pin1 inhibitory mechanism	Refs.
Juglone		Covalently modifies the active site	^9^
Buparvaquone		Covalently modifies the active site	^224^
PiB		Inhibits PPIase activity	^230^
PiJ		Inhibits PPIase activity	^230^
Benzothiophene		Competitively binds to active site	^231, 232^
D-peptide		Competitively binds to active site	^233^
E-peptide		Binds to the catalytic domain	^80^
Phenyl imidazoles		Binds to the active site	^234^
EGCG (epigallocatechin-3-gallate)		Binds to the WW domain and PPIase domain	^235^
ATRA (all *trans* retinoic acid)		Binds to the active site and induces degradation	^36^
*Cis*-locked alkene peptidomimetics		Substrate analogs for Pin1	^225^
Pyrimidine derivative		Covalently binds to Pin1	^236^
Cyclic peptides		Substrate analogs for Pin1	^237^
Imazamethabenz		Combines With Pin1	^226^
6,7,4′-THIF (6,7,4′-trihydroxyisoflavone)		Interacts with the WW domain and PPIase domain	^238^
Rhein		Inhibits Pin1 bind to c-Jun	^239^
KPT-6566		Binds to the PPIase domain and induces degradation	^38^
Thiazole derivative		Substrate analogs for Pin1	^240^
Product-like compound		Substrate analogs for Pin1	^241^
API-1		Binds to the PPIase domain	^227^

**Fig. 3:**
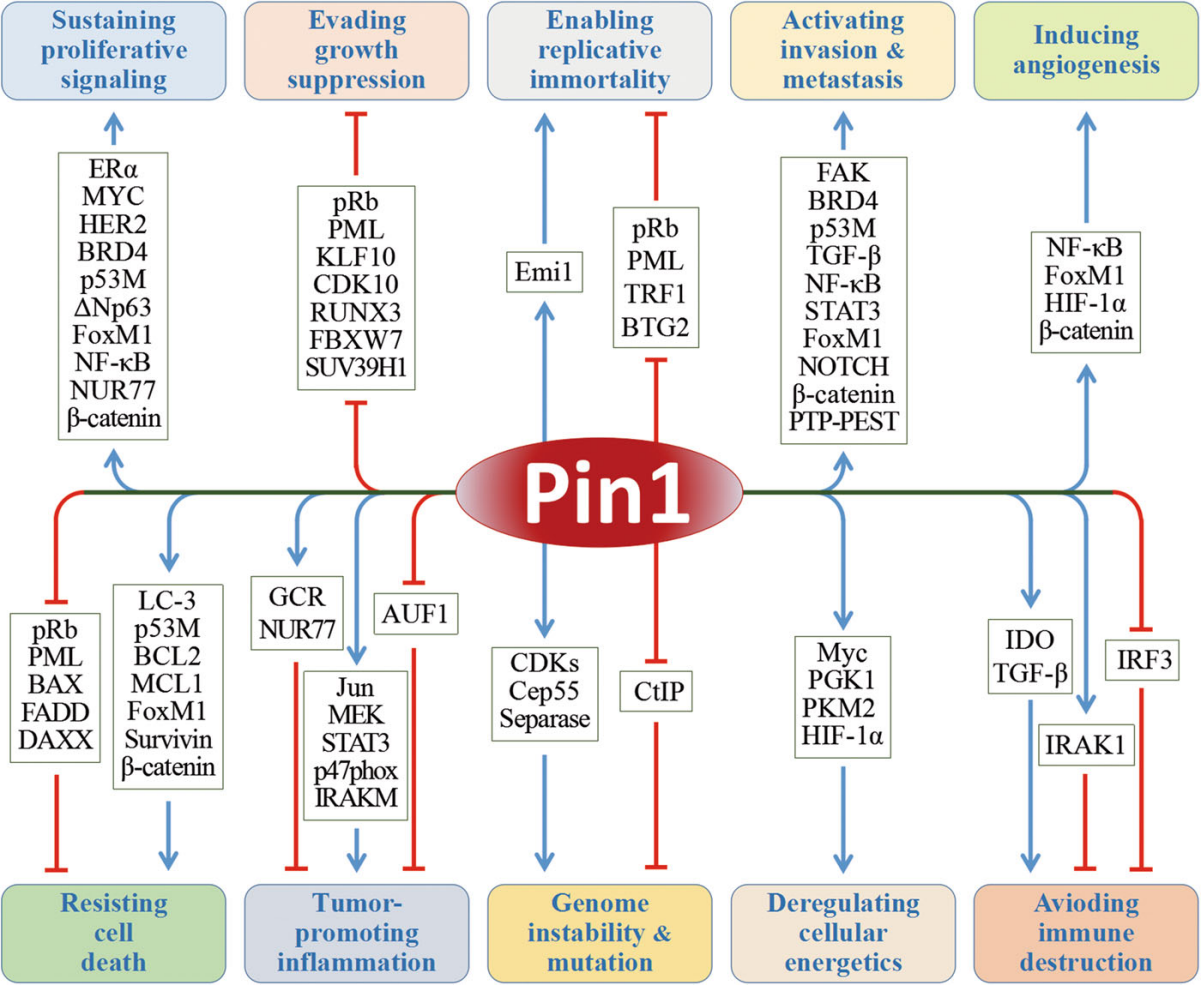
Pin1 extensively participates in multiple cellular processes of cancer. According to the summaries of Hanahan D and Weinberg RA, the hallmarks of cancer contain ten major biocapabilities. Pin1 is highly expressed in the majority of cancers and contributes to all of these aberrant behaviors by dysregulating multiple cancer-driving pathways

According to the existing research, Pin1 upregulates >50 oncogenes or proliferation-promoting factors while inhibits >20 tumor suppressors or proliferation-restraining factors^8,30^ (Fig. 2). However, what are the specific mechanisms of Pin1 in different cancer capabilities? Following the ground-breaking summaries of Hanahan and Weinberg, we have a clearer recognition regarding the hallmarks of cancer. The ten major cancer capabilities are sustaining proliferative signaling, evading growth suppressors, activating invasion and metastasis, enabling replicative immortality, inducing angiogenesis, resisting cell death, evading immune destruction, tumor-promoting inflammation, reprogramming of energy metabolism, and genome instability and mutation^39,40^. Mounts of researches indicated that Pin1 is an active participant of these aberrant processes^8^ (Fig. 3). In this review, we summarized the detailed mechanisms of Pin1 that contribute to these cancer capabilities and certain Pin1-targeted small-molecule compounds that exhibit anticancer activities, expecting to facilitate anticancer therapies by targeting Pin1.

### Pin1 sustains the proliferative signaling

**Fig. 4:**
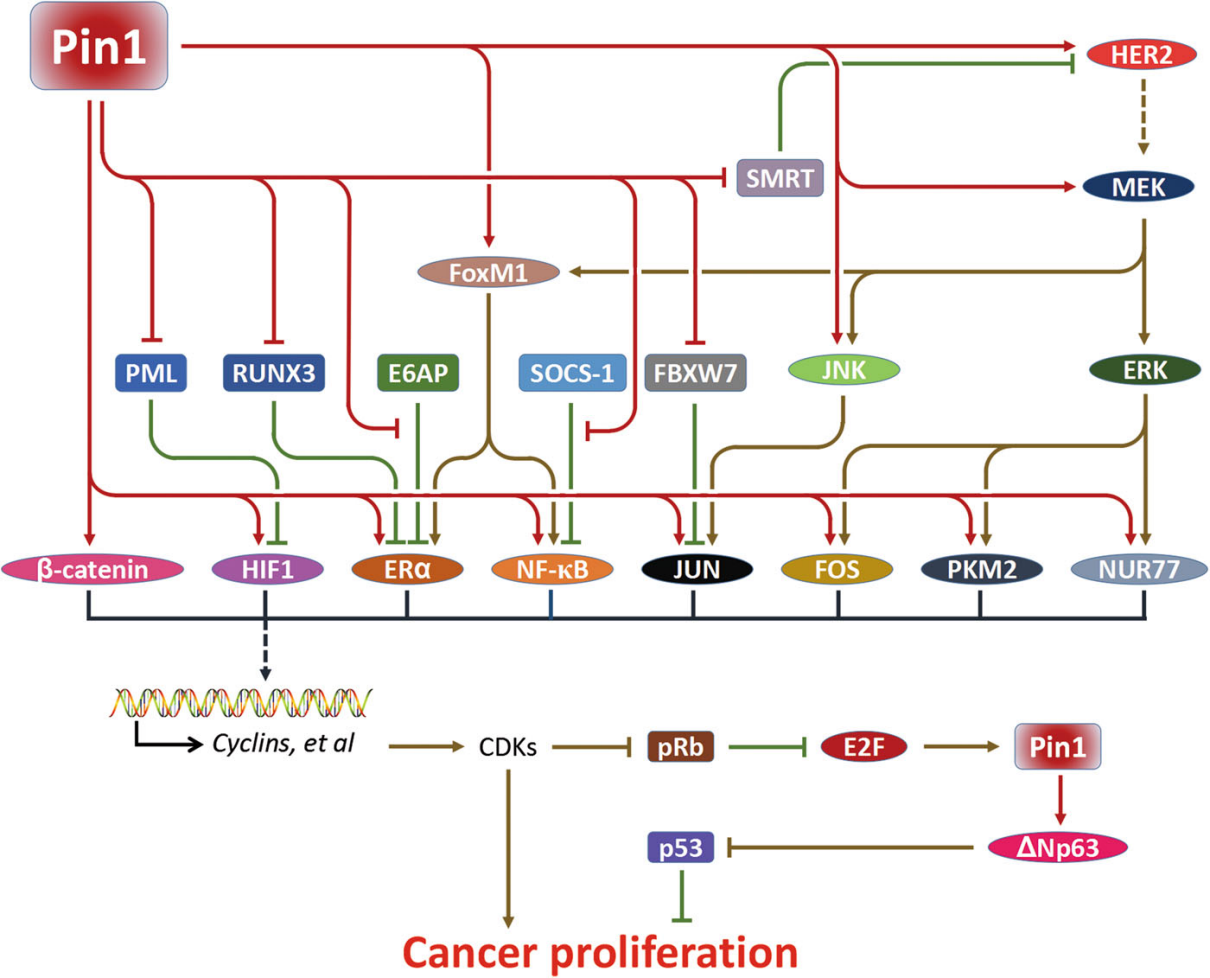
Pin1 facilitates multiple proliferative pathways in cancer. Pin1 upregulates transcription factors, such as β-catenin, HIF1, ERα, NF-κB, JUN, FOS, NUR77, and co-activator PKM2 by enhancing their stability, nuclear translocation, transcriptional activity, and/or activating their upstream regulators in multiple cancers. These transcription factors induce the expression of target genes especially various cyclins to control the cell cycle. Besides, Pin1 is a target of the transcription factor E2F, which is inhibited by pRb. In many cancers, pRb can be phosphorylated and inactivated by CDKs, which facilitates the expression of Pin1. Moreover, Pin1 reverses the p53-mediated growth inhibition by stabilizing ΔNp63. In general, Pin1 facilitates the cancer proliferation through regulating different substrates at several levels

Cell proliferation is strictly regulated by the intracellular and extracellular signals^41,42^, but cancer cells utilize many pathways to sustain proliferation^43,44^. Pin1 was initially identified as a regulator of mitosis^45^ and many subsequent studies showed that it facilitates multiple proliferation-promoting pathways in cancer^46^ (Fig. 4).

Estrogen receptor α (ERα) promotes the proliferation of cancer, especially breast cancer, by regulating the expression of estrogen response element (ERE)-containing genes^47^. Research indicated that Pin1 increases the transcriptional activity^48^, ERE binding affinity^49^, and inhibits the E3 ligase E6AP-induced degradation of ERα in breast cancer^50^. The garlic extract diallyl trisulfide-treated breast cancer cells exhibit a reduced expression of Pin1 along with reduced ERα activity and cell proliferation^51^. Besides, the high expression of Pin1 and HER2 are concurrent in most breast cancers. Pin1 activates HER2 by inhibiting its ubiquitination^52^ and destabilizing its transcriptional corepressor SMRT^53^.

Additionally, activation of the nuclear factor (NF)-κB pathway strongly induces cancer cell proliferation. Pin1 activates the NF-κb pathway by enhancing the nuclear accumulation of RelA/p65, c-Rel, and v-Rel^54,55^. Besides, Pin1 inhibits the E3 ligase SOCS-1-mediated ubiquitination of p65^54^. Pin1-mediated activation of the NF-κB pathway is involved in the proliferation of glioblastoma^56^, endometrial carcinoma^57^, acute myeloid leukemia (AML)^58^, and hepatocellular carcinoma (HCC)^59^.

Furthermore, ΔNp63, an isoform of p63 that lacks an intact N-terminal transactivation domain, is critical for tumorigenesis^60^. Pin1 inhibits the E3 ligase WWP1-induced ubiquitination of ΔNp63 to increase the proliferation of human oral squamous cell carcinoma^61^. Pin1 also stabilizes bromodomain-containing protein 4 (BRD4), a transactivator of multiple oncogenes, to promote the proliferation, migration, and invasion of gastric cancer^62^. Besides, Pin1 upregulates many other proliferation-inducing factors, including β-catenin^63^, FoxM1^64^, XBP1^65^, NUR77^66^, c-Jun^67^, and c-Myc^68^.

Moreover, Pin1 also induces the proliferation of non-tumorous cells, such as pancreatic β cells^69^, hepatic oval cells^70^, and spermatogonial stem cells^71^. The deficiency of Pin1 significantly suppresses the growth of multiple cell types, indicating that Pin1 is a potential target to treat hyperplastic diseases.

### Pin1 downregulates numerous tumor suppressors

The tumor suppressors act as surveillant of multiple cellular processes to prevent cancerization and suppress cancer progression, but cancer cells utilize various mechanisms to surmount these barriers. Research indicated that Pin1 is guilty for the inactivation of numerous tumor suppressors.

When DNA is damaged, the tumor-suppressor retinoblastoma protein (pRb) directly inhibits the transcription factor E2F to arrest the cell cycle^72^. However, pRb is usually inactivated in cancer cells due to reduced expression and/or continuously hyperphosphorylation^73,74^, which partially attribute to Pin1. The insulin-like growth factor 1-stimulated wild-type mouse embryonic fibroblasts (MEFs) exhibit hyperphosphorylated pRb and highly expressed Pin1 simultaneously, but Pin1^−^^/−^ MEFs show a considerably lower level of phosphorylated pRb^75^. Research illuminated that Pin1 promotes CDK-induced phosphorylation^76^ and inhibits PP2A-mediated dephosphorylation^77^ of pRb that subsequently activate E2F and trigger cells into S phase.

The promyelocytic leukemia protein (PML) is another powerful tumor suppressor but always mutant in cancer. Research indicated that Pin1 destabilizes PML to promote the survival and proliferation of breast cancer^78^. Pin1 enhances the E3 adapter KLHL20-induced ubiquitination of PML to promote the proliferation and angiogenesis of prostate cancer^79^. Besides, Pin1 also stabilizes the oncogenic fusion protein PML-RARα in AML^81^. Suppression of Pin1 significantly inhibits the proliferation of breast cancer cells and restores the expression of PML and SMRT^80^.

Additionally, the runt-related transcription factor 3 (RUNX3) acts as an ERα inhibitor in breast cancer^82^. Pin1 decreases the transcriptional activity and increases the ubiquitin-dependent degradation of RUNX3^83^. The E3 ligase FBXW7 suppresses cancer by reducing multiple oncogenes, but Pin1 inactivates FBXW7 by disrupting its dimerization and promoting its self-ubiquitination^84^. Pin1 also downregulates other tumor suppressors, including Kruppel-like factor 10^85^, suppressor of variegation 3-9 homolog 1^86^, and CDK10^87^.

Interestingly, a number of studies indicated that Pin1 increases p53-induced cell senescence and apoptosis^88,89^. However, Pin1 expression is higher in HCC cells with mutant p53 (p53M) compared to wild-type p53 (p53WT), and the deletion of Pin1 significantly reduces the proliferation of p53M HCC cells but not p53WT^90^. More research revealed that Pin1 facilitates the p53M-induced aggressiveness of cancers^33,91,92^, which contributes to a reasonable explanation for why p53 is aberrant in most cancers.

### Pin1 promotes cancer invasion and metastasis

Cancer invasion and metastasis are the leading cause of death in cancer patients. Research revealed that the expression of Pin1 is much higher in the metastatic cancer compared with primary, which reduces the invasion- and metastasis-promoting function of Pin1^93,94^.

The transforming growth factor (TGF)-β pathway inhibits the proliferation but promotes the metastasis of cancer^95,96^. The SMAD proteins are major downstream adapters of TGF-β signal and extensively recognized by WW domain-containing proteins, including Pin1^97,98^. Initial research revealed that Pin1 induces the E3 ligase Smurf-2-mediated degradation of SMADs to suppress the TGF-β signal^99^. However, the later research indicated that Pin1 promotes the TGF-β-induced metastasis of prostate cancer cells^100^. Inhibiting the phosphorylation of SMAD3 reduces the interaction with Pin1 and remarkably suppresses the aggressiveness of breast cancer^101^. Therefore, the function of the TGF-β pathway is complex and Pin1-mediated TGF-β pathway in cancer requires a deeper investigation.

Pin1 also increases the invasion and metastasis of cancer by activating the NOTCH pathway. In breast cancer, Pin1 facilitates the transcriptional activity of NOTCH1 by potentiating its γ-secretase-mediated cleavage^14^. Meanwhile, NOTCH1 induces the expression of Pin1, which consequently form a positive loop to enhance cancer cell transformation^14^. Pin1 also promotes breast CSC self-renewal and metastasis by inhibiting FBXW7-mediated degradation of NOTCH1 and NOTCH4^32^. Besides, Pin1 activates the NOTCH3 signal by enhancing its cleavage and stabilizing its intracellular domain in T cell acute lymphoblastic leukemia (T-ALL) cell lines and mouse models. The deletion of Pin1 markedly decreases the NOTCH-induced invasion of T-ALL cells^102^.

Moreover, PTP-PEST and FAK are two pivotal effectors of the RAS signal, which are involved in tumor metastasis^103^. Pin1 facilitates the interaction of PTP-PEST with FAK to accelerate the Tyr397 dephosphorylation of FAK, which consequently induce the metastasis of numerous cancers^104,105^. Pin1 also promotes the epithelial–mesenchymal transition of MCF-7 cells by increasing the transcriptional activity of signal transducer and activator of transcription factor 3 (STAT3) and recruiting its transcription coactivator p300^106^. Additionally, Pin1 promotes the invasion and metastasis of multiple cancers by activating NF-κB^107^, p53M^108^, β-catenin^63^, and BRD4^62^.

### Pin1 enables the replicative immortality of cancer

After a limited number of cycles, the majority of normal cells enter a nonproliferative but viable state, which called cellular senescence. The cells that continue to divide will face a fatal crisis, which causes the death of most cells, but the minority that passes this barrier will be immortal^39,40^. The mechanisms that control the proliferative barrier include telomere shortening, DNA damage, and mitochondria damage^109–111^. The antisenescence function of Pin1 is widely revealed in vascular smooth muscle cells^112^, cardiac progenitor cells^25^, tendon stem/progenitor cells^113^, fibroblasts^114^, and various cancer cells.

In most cancer, telomerase is reactivated to maintain the telomeric DNA, but telomeric repeat-binding factors (TRFs) prevent its elongation^115^. Research demonstrated that Pin1 elongates the telomere via promoting E3 ligase Fbx4-mediated degradation of TRF1 in multiple cancer cells^116^. Additionally, the early mitotic inhibitor 1 (Emi1) is an inhibitor of the DNA damage-induced senescence^117^. Pin1 promotes the proliferation and suppresses the senescence of several cancer cells by preventing the E3 ligase βTrCP-induced degradation of Emi1^118^. Pin1 also suppresses the senescence-inducing factors pRb and PML in multiple cancers. Pin1 enhances p53-induced senescence and apoptosis; however, inhibiting Pin1 leads to senescence in p53-interfered BJ cells and overexpression of Pin1 reverses the p53 responder BTG2-induced senescence^114^.

Furthermore, the Pin1 deficit contributes to many degenerative diseases, including AD^28^, HD^29^, and PD^27^, all of which are related to aberrant neuronal senescence and apoptosis. Considering the antisenescence function, Pin1 and its substrates are potential targets to treat both degenerative diseases and cancers.

### Pin1 enhances cancer-induced angiogenesis

The angiogenesis is strictly controlled in vivo. However, the rapidly expanded cancer can induce continuous angiogenesis to maintain the sustenance of nutrients and oxygen, as well as the elimination of metabolic waste and carbon dioxide. Abundant evidence illuminated that Pin1 is involved in cancer-induced angiogenesis^119^.

The hypoxia-inducible factor 1α (HIF-1α) strongly induces angiogenesis by promoting the expression of vascular endothelial growth factor (VEGF) in the hypoxia cancer tissue^120^. Studies indicated that the high expression of Pin1, HIF-1α, and VEGF are positively related in TAM-resistant MCF-7 (TAMR-MCF-7) cells^121,122^. Pin1 increases the stability and transcriptional activity of HIF-1α in many cancers^123,124^. PML inhibits HIF-1α-induced angiogenesis both in clear cell renal cell carcinoma^125^ and human umbilical vein endothelial cells^126^, but Pin1 destabilizes PML in multiple cancers. HIF-1α also induces the expression of KLHL20, which cooperates with Pin1 to induce the ubiquitin-dependent degradation of PML^79^. Besides, Pin1 facilitates the NF-κB-induced expression of VEGF in HCC^59^. Additionally, the VEGF-promoting transcriptional factors, such as FoxM1^127^ and β-catenin^128^, are upregulated by Pin1 in numerous cancers.

Inhibition of Pin1 significantly reduces the cancer-induced angiogenesis. Directly suppressing the expression of Pin1 by RNAi inhibits both growth and angiogenesis of prostate cancer^129^. The phosphoinositide-3 kinase (PI3K)/p38 signals increase the expression of Pin1 via activating E2F1^121^. In TAMR-MCF-7 cells, the PI3K inhibitor quercetin^122^ and the E2F1 inhibitor amurensin G^130^ markedly reduced the expression of Pin1, secretion of VEGF, and angiogenesis. In conclusion, Pin1 enhances the angiogenesis of multiple cancers by promoting the expression of VEGF.

### Pin1 facilitates the cell death resistance of cancer

Apoptosis is an important form of programmed cell death, which acts as a natural barrier to prevent cells from developing into cancers^131,132^. However, cancer cells can block the proapoptotic signals and activate antiapoptotic signals to make them survive in cytotoxic stress. Pin1 is a powerful “weapon” of cancer to against apoptosis^133^.

First, Pin1 inhibits the proapoptotic factors. Outer mitochondrial membrane located BAX and BAK induce apoptosis by enhancing the release of cytochrome *c*^134^. In human eosinophils (Eos), Pin1 inhibits the BAX-induced apoptosis by preventing its mitochondria translocation^135^. Besides, the death-associated proteins DAXX and FADD are two critical responders of CD95/Fas-induced apoptosis^136,137^. The Fas signal notably increases the activity and nuclear translocation of FADD by phosphorylating its Ser194 and inhibits Pin1 by phosphorylating its Ser16^138^. However, exogenetic expression of Pin1 maintains the cytoplasmic location of FADD by accelerating its dephosphorylation, which consequently blocks the Fas-FADD pathway^138^. Pin1 also isomerizes DAXX to promote its ubiquitin-dependent degradation in malignant human gliomas^139^. Additionally, DNA damage-induced apoptosis is mediated by many surveillance proteins, such as p53, PML, and pRb. However, PML and pRb are downregulated by Pin1 in numerous cancers. Pin1 enhances p53-induced apoptosis but facilitates the cancer-driving function of p53M.

Second, Pin1 upregulates the antiapoptosis factors. The B-cell lymphoma 2 (BCL-2) family proteins inhibit apoptosis via directly inactivating BAX and BAK depending on their shared BH3 domain^140^. Research indicated that Pin1 enhances the stabilization and cell death resistance ability of BCL-2 and myeloid cell leukemia-1 (MCL-1)^141,142^. Some anticancer drugs, such as sorafenib and amsacrine, induce the apoptosis of cancer cells by reversing the Pin1-mediated stability of MCL-1^141,143^. Pin1 also enhances the survival of cisplatin-treated cervical cancer cells by upregulating Wnt/β-catenin and FoxM1 pathways^144^. In addition, Pin1 upregulates LC-3 to induce protective autophagy, which consequently increases the tamoxifen resistance of breast cancer^145^.

Interestingly, Pin1 increases the antiapoptotic activity of Survivin in HCC^146^, which is opposite in neuroblastoma^147^. In addition to p53 and Survivin, Pin1 also induces cell apoptosis by activating and stabilizing the tumor-suppressor homeodomain interacting protein kinase 2^148^, increasing the mitochondrial translocation of p66Shc^26^, as well as inhibiting the activity of ataxia telangiectasia and rad3 related^149^. However, all of these results were observed in non-tumorous cells.

### Pin1 helps cancer cells to evade immune destruction

With further research on cancer and the immune system, the traditional concept that the immune system prevents tumor initiation and development has been questioned^150^. Cancer cells arising from immunocompetent mice are much more aggressive than that arising from immunodeficient mice^150^. Multiple studies demonstrated that Pin1 participates in the regulation of immune response.

The Toll-like receptors (TLRs) recognize pathogen-associated molecular patterns to initiate the immune response^151,152^. Pin1 is involved in the regulation of the TLR signals. In plasmacytoid dendritic cells, engaged TLR7/TLR9 activate interleukin (IL)-1 receptor associated kinase-1 (IRAK1) and subsequently activated IRAK1 induces the secretion of type I interferon (IFN-αβ) by activating the transcription factor IFN-regulatory factor 7 (IRF7)^153,154^. IRAK1 is autophosphorylated within the activated TLR complex and then Pin1-mediated isomerization induces its liberation from the complex^155^. Pin1-induced overactivation of the TLR-7/TLR-9/IRAK-1/IRF-7 signal contributes to the autoimmune disease systemic lupus erythematosus^156^. In addition, IRF3 is a downstream adapter of TLR3/TLR4 signal that induces the expression of IFN-β in the antiviral response^157^. However, Pin1 reduces the transcriptional activity and promotes ubiquitin-dependent degradation of IRF3 leading to reduced production of IFN-β in poly(I)poly(C)- or RIG-I-stimulated immune cells^158^. Furthermore, the double-stranded RNA-induced expression of IFN-β is significantly lower and the replication of the invading virus is higher in Pin1^+/+^ mice compared with Pin1^–/–^ mice^158^. Both tripartite motif-containing 21 and PML isoform IV stabilize IRF3 and enhance IRF3-mediated production of IFN-β by disturbing the interaction of Pin1 with IRF3^159,160^. Besides, the IRF3-mediated expression of IFN-λ1 is also decreased by the exogenous expressed Pin1^161^. In conclusion, the function of Pin1 in immune regulation is cell-type and pathogen dependent.

Pin1 is involved in the immune escape of cancer. Indoleamine-pyrrole 2,3-dioxygenase (IDO) exhausts local tryptophan to limit the function of T lymphocytes^162^. Research indicated that the cytotoxic T lymphocyte-associated protein 4 (CTLA-4)-stimulated dendritic cells (DCs) produce IDO upon simultaneously activating the PI3K and NOTCH pathways. Pin1 increases the enzyme activity of casein kinase II to abolish the PTEN-mediated suppression of PI3K^163^. Suppressing the NOTCH signal significantly reduces the expression of Pin1 and the CTLA-4-induced IDO production^163^. Besides, TGF-β powerfully inhibits the proliferation and function of multiple immune cells to disorganize the host immune surveillance^164^. In the lung, liver, and cardiac fibrosis mouse models and even in patients, Pin1 increases the expression of TGF-β by stabilizing its mRNA and protein^165–167^. It is clear that Pin1 enhances the TGF-β-induced invasion and migration of cancers, but the Pin1-mediated TGF-β signal in cancer immune escape requires a deeper investigation. In conclusion, targeting the immunoediting checkpoints is an effective strategy to treat cancer^168^ and Pin1 is a potential candidate.

### Pin1 participates in the tumor-promoting inflammation

The release of necrotic cell content and microbe infection induce the inflammatory response by recruiting inflammatory cells into the local microenvironment^169,170^. Research indicated that chronic inflammation is correlative to cancer initiation and progression^171^.

Pin1 is a regulator of inflammatory response. In human neutrophils, NADPH oxidase catalyzes the production of superoxide which subsequently generates reactive oxygen species (ROS) to annihilate ingested microbes^172^. Pin1 promotes the formation of integrated NADPH oxidase by facilitating the membrane translocation of its cytosolic component, p47phox^173,174^. Besides, granulocyte macrophage colony-stimulating factor (GM-CSF) is essential for activation of circulating leukocytes^175^. The 3′-untranslated region of GM-CSF mRNA is abundant in AU-rich elements (AREs) and ARE-binding protein AUF1 negatively regulates its mRNA stability^176^. Pin1 stabilizes GM-CSF mRNA by decreasing the ARE-binding ability of AUF1 in Eos and T lymphocytes^177,178^. Inhibiting Pin1 significantly reduces the production of GM-CSF in allergen-treated rats^179^.

The aberrant Pin1-mediated inflammation contributes to numerous diseases, including cancer. Pin1 enhances the IL-22-induced proliferation and survival of breast cancer cells by activating mitogen-activated extracellular signal-regulated kinases, c-Jun, and STAT3^180^. Pin1 also increases the nuclear translocation of IRAK-M in DCs to enhance the IL-33-induced allergic airway inflammation^181^. Besides, Pin1 is correlated in other inflammatory diseases, including rheumatoid arthritis^182^, periodontitis^183^, diabetes-induced atherosclerosis^184^, nonalcoholic steatohepatitis^185^, and primary biliary cholangitis^186^.

However, research indicates that Pin1 also stabilizes and activates some anti-inflammatory proteins, such as NUR77^187^ and glucocorticoid receptor^188^. Similar to the immune regulation, Pin1 probability plays a dual role in inflammation regulation, but the cancer-promoting function of Pin1 is much more clarified.

### Pin1 regulates the metabolic reprogramming of cancer

The metabolic pattern of glucose in mammalian cells is dependent on the oxygen environment. In aerobic conditions, the common intermediate pyruvate is mainly transferred to the mitochondria and efficiently produces massive ATP through oxidative phosphorylation, while in anaerobic conditions, pyruvate rapidly produces small amounts of ATP via glycolysis in the cytoplasm. The majority of normal cells are fueled by more efficient oxidative phosphorylation except for some special conditions, such as muscle cells in high-intensity exercise. However, the major metabolic pattern of cancer cells is glycolysis even in aerobic conditions, which called “aerobic glycolysis” or “Warburg effect” after its discoverer, Otto Warburg^189^. The “Warburg effect” is beneficial for cancer progression by supplying intermediates for rapid biosynthesis and avoiding cytostatic controls that are induced by activated oncogenes and/or mutant cancer suppressors^190,191^. The mechanisms that result in “Warburg effect” include increased glucose import, aberrant hypoxia response system, and the incapacitation of oxidative phosphorylation^40^. They are correlated with many aberrant signals, including PI3K/Akt/mammalian target of rapamycin^192^, Wnt/β-catenin^193^, HIF-1α^194^, and non-coding RNAs^195^, all of which finally regulate the expression and/or activation of metabolism-associated proteins.

**Fig. 5:**
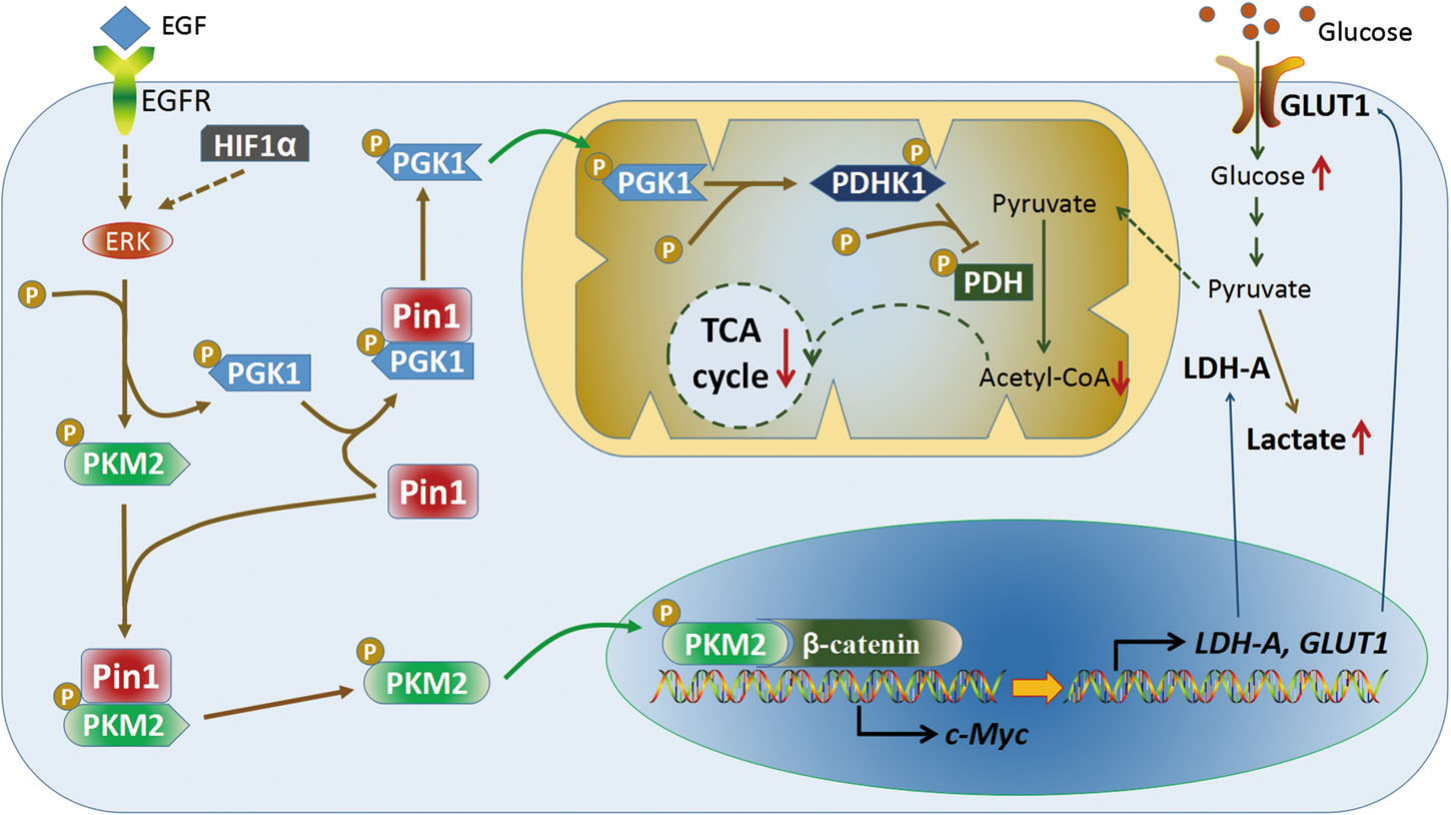
Pin1 is involved in metabolic reprogramming of cancer. “Warburg effect” that first observed by Otto Warburg is an aberrant characteristic of cancer. Pin1 facilitates the glucose metabolic reprogramming of cancer. On the one hand, Pin1 enhances the nuclear localization of PKM2. Nucleus PKM2 acts as a co-activator of β-catenin to promote the expression of LDH-A and GLUT1, thereby enhancing lactification and increasing glucose import, respectively. On the other hand, Pin1 increases the mitochondrial translocation of PGK1. Mitochondrial PGK1 phosphorylates and activates PDHK1, thus inactivating PDH and finally inhibits TCA cycle

Research indicates that Pin1 is a crucial regulator of the “Warburg effect” (Fig. 5). Pin1 increases the nuclear localization of the phosphorylated pyruvate kinase isozyme M2 (PKM2)^196^. The nuclear translocated PKM2 phosphorylates histone H3-T11 leading to the H3-K9 acetylation of target genes. Meanwhile, it also acts as a co-activator of β-catenin to increase the expression of CCDN1 and c-Myc. Subsequently, c-Myc induces the expression of glucose transporter 1 and lactate dehydrogenase A to promote the “Warburg effect”^196,197^. In addition, pyruvate dehydrogenase kinase 1 (PDHK1) suppresses the tricarboxylic cycle by phosphorylating and inactivating the pyruvate dehydrogenase (PDH) complex^198^. Pin1 increases the mitochondria translocation of PGK1 where it activates PDHK1 to facilitate the PDH-inhibited activity of PDHK1^199^. Moreover, Pin1 upregulates many other metabolic regulators directly, such as β-catenin, HIF-1α, and c-Myc. Targeting the metabolic reprogramming process is effective to suppress cancer progression^200^, and these studies revealed that Pin1 is a potential candidate to reverse these dysregulations.

### Pin1 contributes to the genome instability and mutations of cancer

Cytotoxic factors, such as ionizing radiation and DNA topoisomerase II poisons, induce DNA double-strand breaks (DSBs) to trigger cellular senescence and apoptosis^201^. It is effective to suppress cancer by bringing in a mass of DNA damage^202^, but most cancer is insensitive to genome instability and mutations. Pin1 not only suppresses the DAN damage-induced senescence and apoptosis but also contributes the genome instability in cancers.

The mechanisms of DSBs repair include error-free homologous recombination (HR) and fallible nonhomologous end-joining (NHEJ)^203^. CtIP facilitates HR by promoting DNA-end resection^204^. However, Pin1 promotes the ubiquitin-dependent degradation of CtIP to attenuate HR and increase NHEJ, which consequently increase genome instability^205^. Pin1 also interacts with the DSB repair regulators 53BP1 and BRCA1, but there is no further study on them^205^. Besides, it is a promising direction that Pin1 modifies DSB repair through regulating the CDK-mediated DNA-end resection^206^. Meanwhile, rapid proliferation leads to replication-associated DNA damage^207^. Whether the Pin1-promoted proliferation induces DNA damage is also worth of deeper research.

The aberrant mitotic process also contributes to genome instability. Separase promotes sister chromatid paired segregation by cleaving ring-shaped cohesin^208^. Research indicated that the full function of separase requires the Pin1-mediated isomerization at its pSer1126-Pro motif^209^. The centrosome protein 55 kDa (Cep55) is crucial for the formation of midbodies in cytokinesis^210^. Pin1 increases the midbody translocation of Cep55 by facilitating its polo-like kinase 1-mediated phosphorylation at Ser436^211^. Pin1 is also involved in the SEPT9-mediated final separation of daughter cells^212^. Moreover, the overexpression of Pin1 promotes an abnormal centrosome duplication and chromosome instability in breast cancer^213^. Human papillomavirus (HPV) infection also induces genome instability and enhances the malignant phenotype of cervical cancers by promoting aberrant centrosome synthesis^214^. Research indicates that HPV-infected cervical lesions exhibit an elevated level of Pin1^215^, but the relationship of HPV-induced Pin1 and genome instability in cervical cancer requires a deeper investigation.

### Targeting Pin1 is effective to suppress cancer

Targeting a single pathway to treat cancer is challenging because multiple aberrant pathways are involved in cancer progression^39,40^. Moreover, almost all of the current therapies are ineffective to treat CSCs^216^. Therefore, a strategy that simultaneously targets multiple cancer-driving pathways is urgently required. In cancers, Pin1 promotes and suppresses numerous oncogenes and tumor suppressors, respectively. Multiple studies demonstrated that inhibition of Pin1 is effective to suppress the progression of cancers. Aurora kinase A^217^, ribosomal S6 kinase 2^218^ and protein kinase A^19^ abolish the substrate interaction ability of Pin1 by phosphorylating its Ser16 residue in the WW domain. The death-associated protein kinase 1 inactivates Pin1 by phosphorylating Ser71 in the catalytic core of Pin1 that consequently inhibits the centrosome amplification of breast cancer cells^18^ but aggravates neurodegeneration^219^. Pin1-targeted microRNAs (miRNAs), including miR-200b^220^, miR-200c^31^, miR296-5p^221^, miR-370^16^, and miR874-3p^17^, inhibit human cancer progression via directly decreasing the mRNA level of Pin1. However, Pin1 can suppress the miRNA biogenesis by inhibiting exportin-5 (XPO5)^222^. In addition, researchers have discovered and synthesized many Pin1-targeted small-molecule compounds that exhibit anticancer activity (Table 1). ATRA, a currently used target drug for acute promyelocytic leukemia (APL), mechanically combines with the substrate-binding site of Pin1 to inhibit its activation in APL and breast cancer cells^36^. Yang et al. designed a novel slow-releasing, non-toxic, biodegradable, and biocompatible ATRA formulation. Their work showed that this novel formulation exhibits long-term inhibition of Pin1 and is more efficient than the traditional ATRA to suppress HCC cell growth^223^. Juglone, a compound extracted from walnut tree, and its derivative buparvaquone both inhibit Pin1 by covalently modifying its catalytic core^9,224^. Juglone is effective to suppress multiple cancer cells and universally used in Pin1 research. *Cis*-locked alkene peptidomimetics inhibit Pin1 by simulating a substrate of Pin1 and exhibit antiproliferation activities in an ovarian cancer cell line^225^. Imazamethabenz, an imidazoline ketone herbicide, inhibits migration and invasion and induces apoptosis in breast cancer cells via directly combining with Pin1^226^. The Pin1 inhibitor API-1 suppresses HCC development by restoring Pin1-inhibited miRNA biogenesis^227^. Recently, a novel Pin1 inhibitor, KPT-6566, inhibits the PPIase activity and induces the degradation of Pin1 by covalently interacting with its catalytic core^38^. Moreover, inhibiting Pin1 sensitizes many cancer cells to chemotherapy, including HCC to sorafenib^228^, breast cancer to trastuzumab^229^, rapamycin^53^, Taxol and 5-fluorouracil^141^, colon cancer to Taxol^84^, and AML to retinoic acid^81^. Increasing evidence showed that Pin1 is a potential target for cancer therapy. However, the majority of the existing inhibitors lack the required specificity, efficacy, and safety in clinical application.

## Conclusions

The diversity and complexity of cancers are always challenging the treatment. Fortunately, after decades of research, we are uncovering the secret of cancer. Hanahan and Weinberg summarized the common hallmarks of cancer cells, which lets us recognize cancers more clearly. Currently, targeted therapies have applied to the treatment of multiple cancers, which have a higher efficacy and lower side effects than traditional therapies. However, the “smart” cancer cells, especially the CSCs, utilize multiple mechanisms to avoid being eradicated. Therefore, therapies that target common aberrant pathways to block the escape of tumor cells are urgently warranted. Pro-directed phosphorylation is an extensive modification of numerous pathways, which regulate multiple basic cellular processes, including proliferation, differentiation, metabolism, and death. Pin1 is the only known PPIase that mediates the *cis*–*trans* isomerization of pSer/Thr-Pro bond, which highlights its significance in the regulation of Pro-directed phosphorylation.

Pin1 is highly expressed in most cancers, especially CSCs. According to the existing discovery, some regulation loops, including at least Pin1/NOTCH/Pin1 axis^14^, Pin1/pRb/E2F/Pin1 axis^13,72,76^, and Pin1/XPO5/miRNAs/Pin1 axis^31,222^, contribute to the high expression of Pin1. Pin1 is involved in almost every cancer biocapability, suggesting that it is a potential common therapeutic target. There are many Pin1-targeted inhibitors, including the novel structure-based designed compounds, natural extracts, and their derivatives, that exhibit anticancer activity in in vitro, animal models and even in certain patients. Hence, the mechanisms of Pin1-induced cancer progression and targeting Pin1 for cancer therapy are worthy of further investigation. When matured, the extensive clinical applications would benefit many cancer patients.
